# Host Plant-Associated Population Variation in the Carob Moth *Ectomyelois ceratoniae* in Iran: A Geometric Morphometric Analysis Suggests a Nutritional Basis.

**DOI:** 10.1673/031.007.0201

**Published:** 2007-01-16

**Authors:** Fariba Mozaffarian, Alimorad Sarafrazi, Gadir Nouri Ganbalani

**Affiliations:** ^1^ Insect Taxonomy Research Department, Iranian Research Institute of Plant Protection, Tehran, 19395-1454, Iran; ^2^ Insect Taxonomy Research Department, Iranian Research Institute of Plant Protection, Tehran, 19395-1454, Iran; ^3^ Faculty of Agriculture, Moghadas-e Ardebili University, Ardebil, Iran

**Keywords:** population variation, host, carob moth, geometric morphometrics

## Abstract

The carob moth, *Ectomyelois ceratoniae* (Zeller, 1839) (Lepidoptera: Pyralidae), is the most important pest of pomegranate in Iran. As it has been rarely recorded on other host plants, control methods have mostly been focused on its populations on pomegranate. In this study, shapes and sizes of wings were compared in populations on 4 host plants (pomegranate, fig, pistachio and walnut) using a landmark-based geometric morphometric method, and analysis of partial warp scores and centroid sizes. The results showed significantly smaller wing size in populations on pomegranate and a significant host plant-associated shape difference among populations as a consequence of allometric growth. This suggests that the wing size and shape differences among test populations may not have a genetic basis and could happen because of differences in the nutritional content of host plants. The results of the analysis suggest that the female carob moth lays her eggs on host plants that provide suitable conditions for hatching. The larger size of moths on hosts other than pomegranate showed that some host plants such as fig, pistachio and walnut can provide for increased stored nutritional reserves by larvae that may result in more successful over-wintering and higher fecundity in adults. This suggests that in spite of the more extensive activity of carob moth on pomegranate in Iran, populations on other host plants can have an important effect on expanding pest population sizes in following years which should be considered in control methods.

## Introduction

It is believed that nominally polyphagous species of herbivorous insects sometimes are comprised of multiple morphologically similar biological species with more specialized appetites (Adams and Funk 1997). If host plant species constitute different selective regimes to herbivorous insects, genetic differentiation and host plant-associated local adaptation may occur (Ruiz-Montoya et al. 2003). Successful control of any pest is based on correct identification, and inability to recognize distinct populations can have drastic and costly consequences for pest management (Menken and Ulenberg 1987). Hence the existence of host-associated populations has been examined in several insect pests (Downie et al. 2001; Abdullahi et al. 2003; Sarafrazi et al. 2004). On the other hand, polyphagous insects have the advantage that they can feed on different hosts that provide different nutritional resources. In fact natural selection can result in evolution of both specialists and generalists (Lazarevic et al. 1998). The evolution of polyphagy and its benefits have been studied in a number of insects including the locust (Sword and Dopman 1999), and the whitefly (Bezerra et al. 2004).

The pomegranate is one of the most ancient edible fruits. According to Shakeri (2004), up to 60412 hectares of this fruit have been cultured in Iran in recent years. The high yield and quality of pomegranates in Iran has made it an important export commodity. The most important pest on this fruit is carob moth, *Ectomyelois ceratoniae* (Zeller, 1839) (Lepidoptera: Pyralidae), the larva of which feed inside the fruit and highly affects fruit quality. This moth is commonly found in pomegranate orchards in Iran, but there are only a few records of its damage on other host plants such as fig (Shakeri 1993) and pistachio (Mehrnejad 2002); it is a major pest on citrus, date, almond and etc. in the other countries (Morton 1987; Alrubeai 1987; Warner et al. 1990; Van den Berg 1995; Tous and Ferguson 1996; Mesbah et al. 1998; Bouka et al. 2000). The most recommended control method for this pest in Iran is by collecting and destroying infected pomegranates at the end of growth season that eliminates over-wintering sites (Behdad 1991). This control method has also been used for controlling the pest on other fruit such as macadamia (Van den Berg 1995). Biological control (Nasrollahi et al. 1998), staffing the pomegranate fruit neck (Mirkarimi 1996), and removing flags (Shakeri 2004) are other methods that have been described. The two latter methods also remove hatching sites.

**Figure 1.**
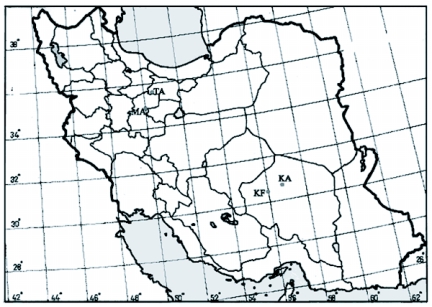
Distribution of collecting sites of host plant associated populations of *Ectomyelois ceratoniae* in Iran. Fi = fig; Pi = pistachio; Po = pomegranate; Wa = walnut. KA = Kerman province, Chatrood; KF = Kerman province, Rafsanjan; MA = Markazi province, Saveh; TA = Tehran province, Eshtehard.

The null hypothesis of the current study is that *E. ceratoniae* populations on different hosts are genetically the same. In this case, adult *E. ceratoniae* oviposits on any available host plant as soon as adults eclose in early May until the end of autumn. If so, the existence of the pest on different hosts during its life cycle can have an effect on its survival. Knowledge of this subject may provide a better view in the pest control.

**Figure 2.**
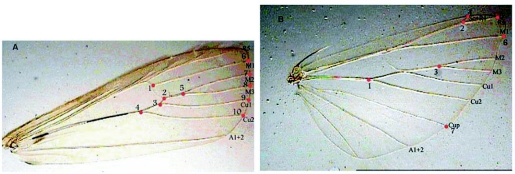
Distribution of landmarks on A) forewing and B) hindwing of *Ectomyelois ceratoniae*. Nomenclature of wing venation is burrowed from Borror *et al*., 1989.

If the null hypothesis is rejected, populations of *E. ceratoniae* show a degree of host plant fidelity that causes divergence of those populations. In this case, adult *E. ceratoniae* oviposits on prefered host plants as soon as suitable fruits are available. After completing one or more generations, the larvae or pupae over-winter in infected fruits. This situation may lead to reduction of gene flow between populations as occurs in the process of sympatric speciation (Walsh 1864; Brues 1924; Thorpe 1930 and 1945; Menken and Ulenberg 1987).

## Materials and Methods

### Preparing specimens for study

Specimens were collected from several provinces in Iran. From the provinces of Kerman: from the city of Rafsanjan on pistachio (KF, Pi) and Chatrood on pomegranate (KA, Po). From the province of Markazi: from the city of Saveh on fig (MA, Fi) and pomegranate (MA, Po). From the province of Tehran: from the city of Eshtehard on walnut (TA, Wa) (Figure 1, Table 1). Specimens were collected from pomegranate in July and August, and from fig, pistachio and walnut in September and November.

**Table 1.**
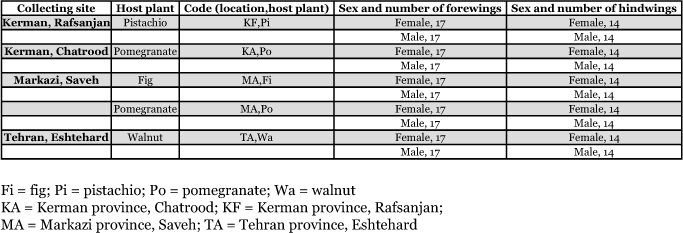
List and code of collecting sites and host plants, and the number of male and female carob moths.

During the collections, infected fruits were collected and the larvae inside them were reared to adulthood in the laboratory. Wing slides were prepared from adults and examined using a dissecting microscope and a CCD video camera. To detect the genetic or environmental basis of host selection, some of the adults that emerged from infected pomegranates from Saveh, were transferred to pistachio and walnut to produce new generations that could be examined for differences between them. 17 forewings and 14 hindwings were obtained from individual moths reared on walnut (male) and pistachio (male and female).

### Geometric morphometric and statistical analysis

10 landmarks on the forewing and 7 landmarks on the hindwing were chosen (Figure 2), and their Cartesian coordinates were digitized by tpsDig (Rohlf 2003a). Landmark data provide some information such as the orientation, rotation and scale of the specimen. The non-shape information was held constant mathematically to remove non-shape variation (Rohlf and Slice 1990). Then using generalized procrustes analysis, all specimens were superimposed so that all homologous landmarks were as close as possible (Rohlf and Slice 1990). Points provided by aligned specimens were projected to the tangent space of a Kendall shape space (Kendall 1984; Rohlf 1999; Slice 2001), so that distances between specimens approximate the procrustes distance between the corresponding pairs of landmark configurations (Adams et al. 2004). Shape variables of geometric morphometric (partial warp scores) were then generated using the thin-plate spline equation (Bookstein 1991). Such variables provide a quantification of overall shape that can be used in conventional statistical analysis, and preserve the geometry of anatomical structure, as well as present mean forms, shape attends and its covariation with other variables (Adams and Rohlf 2000). Uniform components that describe shape changes such as infinitive scale stretching or compression (Bookstein 1996; Rohlf and Bookstein 2003) were calculated by uniform equation (Bookstein 1989, 1991, 1996). In this study uniform components of shape variation were appended as additional columns in the matrix of partial warps (**W** matrix) as suggested by Rohlf et al. 1996. Centroid sizes (the square root of the sum of squared distance of set of landmarks from the center of gravity or the square root of the sum of the variances of the landmarks about that centroid in x and y-directions) as a size measure of any specimen (Slice et al. 1996) were calculated and used as variables in univariate statistical analysis for comparing the size of specimens (Adams and Funk 1997). The above analyses were performed by tpsRelw (Rohlf 2003b).

**Figure 3.**
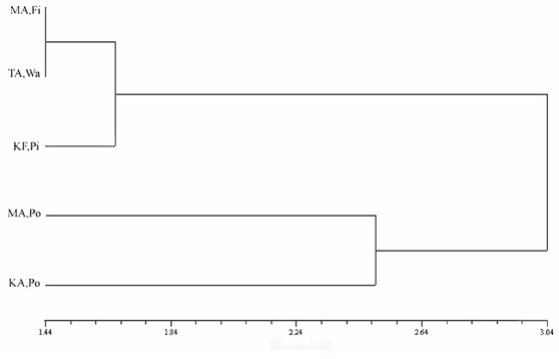
Cluster analysis of tested populations of *E. ceratoniae* showing morphologic distances obtained from the UPGMA method (forewing, female). Fi = fig; Pi = pistachio; Po = pomegranate; Wa = walnut. KA = Kerman province, Chatrood; KF = Kerman province, Rafsanjan; MA = Markazi province, Saveh; TA = Tehran province, Eshtehard.

**Table 2.**
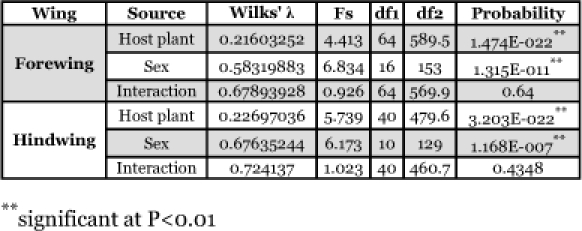
Two-way MOANOVA on **W** matrix of host plant associated populations of *Ectomyelois ceratoniain* Iran.

A two-way MANOVA was performed to find significant differences among test populations and sexes, and to test whether the differences are the same in each sex. Morphologic distances among test populations were computed and the resultant distance matrixes were also subjected to cluster analysis by the unweighted pair group method to show similarity among test populations. In order to minimize the geographic effects (distances and barriers), and compare host plant associated populations, sympatric or quasi-sympatric populations on different host plants were compared: fig from Markazi province (Saveh, MA-Fi) versus pomegranate from the same site (MA-Po), pistachio from Kerman province (Rafsanjan, KF-Pi) versus pomegranate from the same province (Chatrood, KA-Po), Walnut from Tehran province (Eshtehard, TA-Wa) versus pomegranate from Markazi province (Saveh, MA-Po). Critical α were calculated using the Bonferroni method (Sokal and Rohlf, 1995), hence the error rate of 0.05 was divided into the number of comparisons in any sex and wing (3) and the critical α equal to 0.0167 was obtained.

**Table 3.**
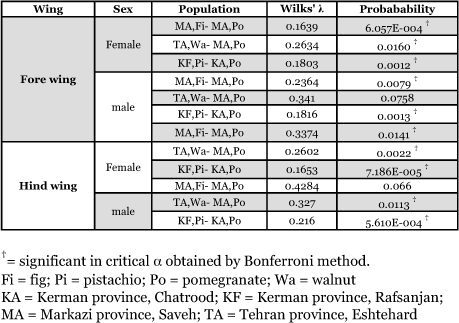
Comparing shape on sympatric or quasi-sympatric host plant-associated populations of *Ectomyelois ceratoniae* in Iran.

To find any isometry in size variation between populations, analyses of allometry among known groups were performed. If rejected, there would be another question to be answered: Do the populations vary in different allometric trajectories? In other words, “Would shape vary significantly between populations if size were held constant?” Regression of shape on size was performed to find any allometry among pair-wise compared populations and then MANCOVAs were designed in any pair of compared populations using the method of fitting constants (Rohlf 2000) to compare allometric slopes and shape when size is held constant.

To determine whether the size of wings in known groups varies significantly, ANOVAs on matrix of centroid size were performed. Statistical analyses were performed using NTSYS-pc (Rohlf 1998) and MINITAB (Minitab Inc. 2000).

## Results

Two-way MANOVA showed the existence of significant differences among wing shapes in host plant associated and sexual populations, however interactions between them were not significant (Table 2). The cluster analyses resulted in similar plots for female and male fore and hindwings as shown in Figure 3. Comparison of wing shape in geographically closed associated populations showed significant differences in nearly all comparisons at a critical α of 0.0167 (Table 3).

**Table 4.**
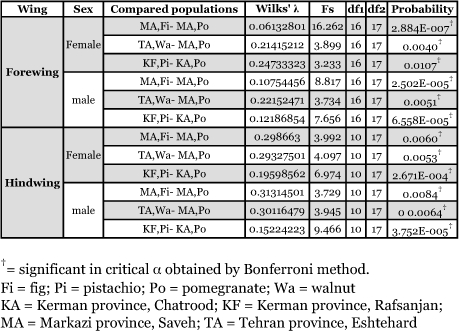
Regression of shape on size in host plant populations of *Ectomyelois ceratoniae* in Iran.

Regression of shape on size in the above comparisons showed significant allometric growth between sympatric or quasi-sympatric host plant-associated populations (Table 4). According to the results of designed MANCOVAs all compared populations do not have significantly different allometric slope (columns marked a in Table 5). Hence the allometric trajectories of any compared populations are parallel with each other. Comparing wing shape in constant size showed non-significant differences (columns marked b in Table 5), therefore, the shape variation in compared populations had the same allometric trajectories. The above analyses showed that significant shape differences among the compared populations shown in Table 3 are the allometric consequence of size difference among them.

**Figure 4.**
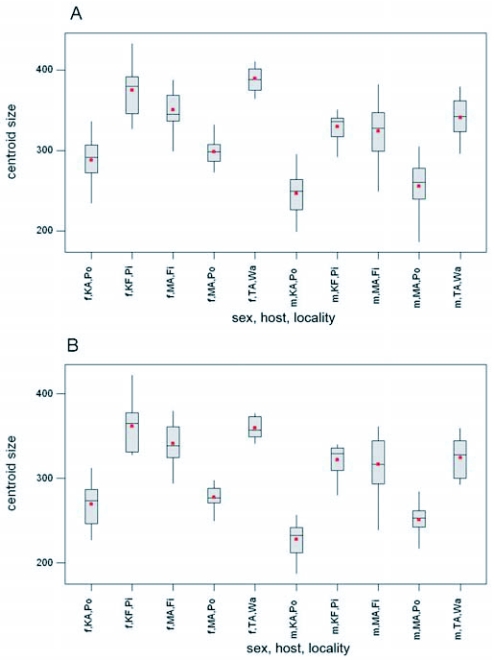
Comparison of size in host plant associated populations in both sexes of *Ectomyelois ceratoniae* in Iran. f: female and m: male populations. A) forewing, B) hindwing. Fi = fig; Pi = pistachio; Po = pomegranate; Wa = walnut. KA = Kerman province, Chatrood; KF = Kerman province, Rafsanjan; MA = Markazi province, Saveh; TA = Tehran province, Eshtehard.

**Figure 5.**
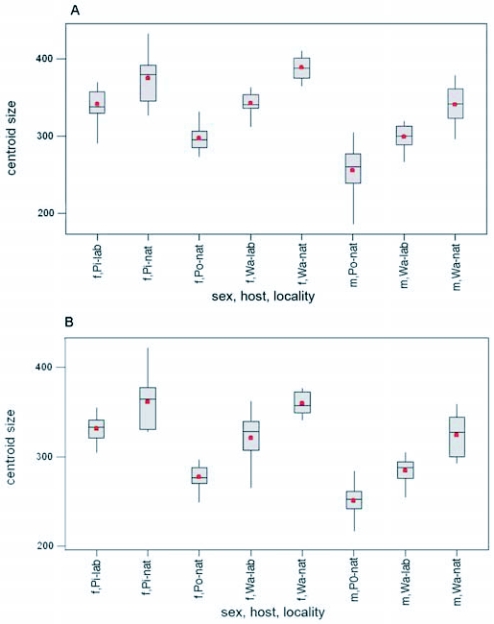
Comparing size of wings between populations reared on different host plants. A) fore wing, B) hind wing. Plant abbreviations: Po = pomegranate, Pi = Pistachio, Wa = Walnut, nat = in nature, lab = in laboratory.

Comparing centroid sizes of host plant associated populations showed significant differences between them (forewing, female: F = 51.92 P = 0.000, forewing, male: F = 46.36 P = 0.000, hindwing, female: F = 49.41 P = 0.000, hindwing, male: F= 49.33 P = 0.000), and in all comparisons pomegranate associated populations had smaller wings than other host plant populations (Figure 4). Comparing wing size of test populations reared on natural and laboratory-reared host plants showed similar results. Although pomegranate-associated populations that were reared on pistachio and walnut in laboratory had smaller wing size than those associated with the same host plants in nature, they had larger wing size than the populations of their parents on pomegranate in nature (Figure 5).

**Table 5.**
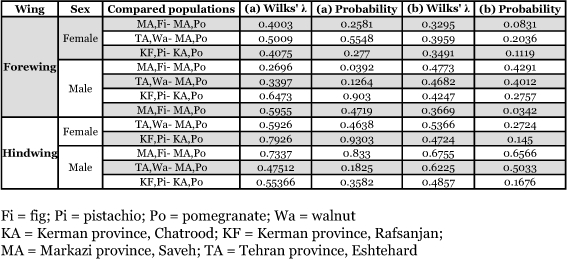
Comparison of allometric slope (a) and shape in constant size (b) of host plant populations of *Ectomyelois ceratoniae* in Iran

## Discussion

Cluster analyses of morphologic distances showed that wing shape within populations feeding on pomegranate is more similar than those feeding on other host plants. The similarity patterns of populations on pomegranate from Saveh (MA, Po) are more similar to those from Chatrood (KA, Po), which are about 770 Km apart, than those on fig from the same site and also on walnut from Eshtehard which is much closer (Figure 1). Since natural variation in populations of a species is the product of interactions between their genetics and environment (Ruggiero et al. 2004), the resulting wing shape similarity may occur because of host plant fidelity, similar to what found in the fruit fly *Rhagoletis pomonella* (Feder et al. 1990 and 1993), and the gelechid moth *Yponometua* sp. (Menken et al. 1992). Alternatively, environmental conditions such as similar nutrition, or other environmental factors, could cause this effect. In the latter case, *E. ceratoniae* has the ability to exploit alternative host plants, which helps insects perform relatively well under new conditions (Milne and Walter 2000). This phenomenon has been detected in many species; Abdullahi et al. 2003 found that except for cassava-associated populations of the whitefly *Bemisia tabaci*, populations on other host plants were polyphagous and did not show significant differences. Downie et al. 2001 found no evidence for differentiation of aphid *Daktulosphaira vitifoliae* populations on two different host plants and McCall et al. 2004 found little genetic variation within host plant-associated populations of a cecidomyiid fly.

Comparison of pair-wise geographically close populations on different host plants revealed significant shape variation in nearly all comparisons. Analyses of wing size showed that populations feeding on pomegranate have smaller wings than those feeding on the other host plants, even smaller than their own offspring that were reared on other host plants in laboratory. Allometric analyses showed allometric growth in test populations, i.e.; variation in size of specimen was accompanied with variation in shape and sympatric or geographically close populations on different host plants varied in the same allometric trajectory. Hence, significant shape variations between compared populations occurred because of the allometric consequence of size change, rather than having a genetic basis. A lack of genetic basis of variations between host associated populations of *E. ceratoniae* was confirmed by further molecular analysis carried out by the authors (unpublished data). The molecular data obtained using 6 amplified fragment length polymorphism (AFLP) primer combinations showed high levels of variation within populations of *E. ceratoniae*, but non-significant genetic distances among sympatric host associated populations.

*E. ceratoniae* which emerges from early May in Iran, preferably attacks pomegranate first. Apparently this fruit provides suitable conditions for oviposition. This may be because of the physical condition of pomegranate neck that protects the eggs and leads larvae to the inside of the fruit. After completing some generations on pomegranate, as soon as other host plants provide suitable conditions for laying eggs, such as the grooves and tracks that occur on pistachio (Mehrnejad 2002), some individuals may attack those host plants in addition to pomegranate. Our results showed that moths emerging from pomegranate have smaller wing size than those from alternate hosts. This increase in wing size is accompanied with wing shape change and may predict increasing body size. Since the compared populations were sympatric or quasi-sympatric, the significant differences in size among populations cannot be the result of location conditions. However, phenological effects could be involved. As it was mentioned before, the populations on pomegranate and on other hosts were collected in second half of summer and first half of autumn respectively. Since populations on pomegranate had smaller wing size in spite of longer photoperiod and warmer temperatures, the size difference cannot be explained by phenological factors. Also, the results of rearing pomegranate populations on other hosts (Figure 5) showed that natural populations had larger wings on any host compared to those reared in the laboratory in any sex, as was expected. However the populations reared on alternative hosts (pistachio and walnut) had larger wing size than their natural parent populations that were collected on pomegranate. Hence, the significant size differences may be the result of host-based effects. Fig, pistachio and walnut may have nutritional factors that promote the growth of larvae and adults that appear later.

It has been shown that the adult body size of some insects such as cerambycid beetles (Andersen and Nilssen 1983; Hanks et al. 2005), dung beetles (Moczek 1998), Japanese horned beetles (Karino et al 2004) and mosquitoes (Telang and Wells 2004) is determined by nutritional conditions or host plant quality during the larval period, and this larger body size can affect their fitness (Clutton-Brock 1988). Large females often have greater longevity and higher fecundity, and larger males have enhanced mating success (Butler and Day 1984). Rodrigues and Moreira (2002) showed that body size increases fecundity in female of *Heliconius erato*, and it was shown that large flies have some advantages compared to small ones such as having higher mating fertilization and reproductive success (Simmons and Parker 1992) even if it is not correlated with genetic variation (Thomas and Barker 1993). In our study, increased storage of nutritional reserves by larvae may result in an increase population size in next generation and may increase successful over-wintering in larvae. Therefore some host plants, such as fig, walnut, and pistachio and perhaps some other host plants, can have more important roles in successful over-wintering of *E. ceratoniae* than infected pomegranates of the previous year. Hence, infected pomegranates may not be the only sites for over-wintering of *E. ceratoniaes* and collecting them may not effectively prevent over-wintering. Our observations while collecting specimens showed that high levels infestation by *E. ceratoniae* on host plants other than pomegranate were not seen. However, considering that our results suggest that infestation of other host plants is likely, it is necessary that control measures should not focus only on pomegranate, as alternate host plants could be important sites for increasing *E. ceratoniae* population in the future.

## Abbreviations

Fi: Fig
KA: Kerman province, Chatrood
KF: Kerman province, Rafsanjan
MA: Markazi province, Saveh
Pi: Pistachio
Po: Pomegranate
TA: Tehran province, Eshtehard
Wa: Walnut
